# Nerve Repair Strategies in Iatrogenic Inferior Alveolar Nerve Injuries: A Systematic Review

**DOI:** 10.7759/cureus.86657

**Published:** 2025-06-24

**Authors:** Jhosue A Olmedo Verdezoto, Emilio J Ponce Basurto, Carlos J Campaña Alcivar, Washington D Rivera Piguave, Samael V Rivera Espinoza, Lisseth E Fernández Oña

**Affiliations:** 1 Outpatient Care, Ministerio de Salud Pública, Quito, ECU; 2 Outpatient Care, Ministerio de Salud Pública, Chone, ECU; 3 Outpatient Care, Patronato Municipal de Inclusión Social de Santo Domingo, Santo Domingo de los Tsáchilas, ECU; 4 Outpatient Care, Ministerio de Salud Pública, Lago Agrio, ECU; 5 Outpatient Care, Ministerio de Salud Pública, Santo Domingo de los Tsáchilas, ECU

**Keywords:** ian, iatrogenic injury, inferior alveolar nerve, microsurgery, nerve repair strategies

## Abstract

The inferior alveolar nerve (IAN) is an essential branch of the trigeminal nerve in the mandible. It provides the nerve supply to the lower lip, chin, and teeth. Despite various nerve repair strategies having been explored to enhance functional recovery, the optimal approach remains unclear. This review aims to identify evidence-based surgical repair techniques for managing iatrogenic IAN injuries. The systematic review inclusion criteria were restricted to English-language full-text articles reporting clinical outcomes of nerve repair interventions in iatrogenic IAN injury. Comprehensive literature searches were performed in PubMed, Embase, and Cochrane Library using relevant keywords and Boolean operators from 2000 to 2025. The review was conducted according to PRISMA guidelines. The methodological quality of included studies was appraised using the Joanna Briggs Institute (JBI) critical appraisal tools for randomized controlled trials (RCTs) and cohort studies.

The systematic review examined outcomes of nerve decompression, neurorrhaphy, and grafting surgeries, with populations ranging from six to 167 patients and average ages between 36 and 56 years, predominantly female. Common causes of inferior alveolar nerve (IAN) injury included third molar extractions (37.6% of cases), dental implant placement, endodontic treatments, and orthognathic surgeries, while non-surgical causes like trauma, delayed intervention, anatomical variations, and patient-related factors (e.g., age) also influenced outcomes. Surgical techniques such as decompression, neurolysis, autogenous nerve grafts (e.g., sural nerve), and neurorrhaphy were compared, with decompression and neurolysis showing 85% and 75% success rates, respectively, for minor injuries. In comparison, grafts and neurorrhaphy achieved 87.3% and 88.9% success rates for severe cases. Recovery times and outcomes were better with early intervention (<12 months), and factors like age, surgical precision, and technique (e.g., piezosurgery reducing thermal damage) significantly impacted results. Complications included donor site morbidity (e.g., temporary numbness) and incomplete recovery in delayed cases, underscoring the need for timely, skilled intervention. The review highlighted that early surgical intervention is a key determinant in achieving optimal results for IAN injury treatment. Results indicated that the selection of the surgical nerve repair strategy depends upon the patient factors, type of injury, and severity. Moreover, when surgical strategies are performed within two months of the incident, this greatly improves the chances of regaining functional sensory recovery in the affected area. The highest success rate was found with direct neurorrhaphy and sural nerve grafting among a variety of surgical techniques, most noticeable in clean injuries. The decompression and neurolysis techniques showed modest effectiveness, although they worked better in a few chronic compression cases. New approaches like using piezosurgery and computers to help with surgical navigation improve safety and lessen the injury caused by surgery. Nevertheless, nerve grafting usually leads to some short-term and easy-to-handle donor site morbidity. The lack of standardized outcome reporting and direct comparative randomized controlled trials with homogenous assessment tools limits the generalizability of the findings and recommends further validation.

## Introduction and background

In dental and oral surgeries, especially during wisdom tooth removal, placing dental implants, and treating bite problems, the inferior alveolar nerve (IAN) is damaged more than any other nerve [1,2]. For iatrogenic IAN injuries, the mechanism can be pressure, stretching, a partial cut, or thermal damage, which influences their pathophysiology [3]. Due to these injuries, people may suffer from abnormal sensory problems like paresthesia, dysesthesia, and severe pain, which seriously reduce their daily lives [4]. Sunderland's method is popular for grading nerve injuries, and surgeries are often considered for types III-V [5]. Performing the repair soon after the injury, within three to six months, results in better recovery of sensation than repair done later on, especially when the damage is partial. Improvement may be seen in up to 85-90% of patients within six to eight weeks [6].

The outcome of iatrogenic inferior alveolar nerve (IAN) injuries depends on how severe the damage is, how quickly the problem is treated, and the specific course of treatment. Minor injuries usually heal without treatment, but significant injuries that involve nerve tissue can become serious and often require surgery, but have a poorer outcome [7]. Patients typically recover their senses more effectively if diagnosis and treatment start within three to six months [8]. Persistent tingling or pain, such as dysesthesia or neuropathic pain, may suggest a poorer outcome. Improving nerve function with surgery is promising, even if total recovery is rarely possible, which means early and customized care is essential [9].

Iatrogenic inferior alveolar nerve injuries should be treated according to how severe the injury is, how early it was recognized, and individual patient details. In the case of mild neurapraxia injuries, treatment is generally conservative, involving corticosteroids and nonsteroidal anti-inflammatory drugs (NSAIDs), with monitoring [10]. In severe axonotmetic or neurotmetic injuries, the priority is microsurgery, with closed repair for no-tension cuts and nerve grafting (using sural or great auricular nerves) needed for larger gaps [11]. Although microsurgery using direct neurorrhaphy, autografting, and conduit-assisted repair has shown results, non-surgical options like drugs, lasers, and nerve protectors are now being considered [12]. Microsurgical repair is still the primary treatment, often needing coaptation or grafts [13]. Nerve conduits such as those made from bioengineered materials or enhanced with growth factors might replace autografts, as they lower morbidity in the area where the donor graft is obtained [14].

Despite having more invasive dental treatments worldwide, infections of the IAN are still a pressing problem, calling for reliable techniques to fix and restore nerve functions. Given the heterogeneity in treatment approaches and outcomes reported in the literature, this systematic review adheres to rigorous methodology to ensure reliable and unbiased conclusions. This review aims to establish evidence-based guidelines for managing iatrogenic IAN injuries by synthesizing data from clinical studies. Ultimately, this review seeks to improve clinical decision-making, reduce patient morbidity, and pave the way for innovative therapeutic advancements in nerve repair strategies.

## Review

Methodology

This systematic review was conducted while following the Preferred Reporting Items for Systematic reviews and Meta-Analyses (PRISMA) guidelines [15]. The research question was formulated using the Patient/Population, Intervention, Comparison, and Outcome (PICO) framework in Table 1 [16].

**Table 1:** PICO framework along with related keywords and controlled vocabulary MeSH - Medical Subject Headings; PICO - Patient/Population, Intervention, Comparison, and Outcome

Concepts	Text words	Controlled vocabulary
Population/Problem - Adults with iatrogenic inferior alveolar nerve (IAN) injury due to dental or maxillofacial procedures	"Inferior alveolar nerve injury" OR "IAN injury" OR "Iatrogenic nerve injury" OR "Dental nerve injury" OR "Nerve injury after tooth extraction" OR "Nerve damage after implant"	"Inferior Alveolar Nerve"[MeSH] OR "Iatrogenic Disease"[MeSH] OR "Peripheral Nerve Injuries"[MeSH] OR "Dental Implantation"[MeSH] OR "Tooth Extraction"[MeSH] OR "Orthognathic Surgery"[MeSH]
Intervention - Direct surgical nerve repair techniques	"Microsurgical repair" OR "End-to-end neurorrhaphy" OR "Nerve graft" OR "Nerve conduit" OR "Nerve tube" OR "Nerve coaptation" OR "Surgical decompression" OR "Tension-free repair"	"Nerve Transfer"[MeSSH] OR "Peripheral Nerve Repair"[MeSH] OR "Nerve Grafting"[MeSH] OR "Surgical Procedures, Operative"[MeSH] OR "Microsurgery"[MeSH]
Comparison - No intervention, delayed surgery	"No repair" OR "Delayed repair" OR "Different nerve repair technique" OR "Conservative treatment"	"Delayed Repair"[MeSH] OR "Treatment Outcome"[MeSH] OR "Conservative treatment"[MeSH]
Outcomes - Functional nerve recovery, sensory improvement, patient satisfaction	"Sensory recovery" OR "Paraesthesia improvement" OR "Dysesthesia resolution" OR "Nerve function restoration" OR	"Sensory Thresholds" [MeSH] OR "Paraesthesia" [MeSH] OR "Nerve Regeneration" [MeSH]

Research Question

What are the outcomes of surgical repair strategies for iatrogenic inferior alveolar nerve (IAN) injuries in adults following dental or maxillofacial procedures?

Search Strategy and Search Terms

Searches were conducted using combinations of keywords and controlled vocabulary related to "inferior alveolar nerve injury", "IAN injury", "surgical repair techniques", "microsurgical repair", "sensory recovery", "nerve conduit", and "neurorrhaphy". Boolean operators "AND" and "OR" were used to connect relevant terms across databases, including PubMed, Embase, and the Cochrane Library. The search focused on text words and controlled vocabulary (e.g., Medical Subject Headings (MeSH) terms) to ensure comprehensive coverage. Limiters were applied to retrieve only open-access, full-text articles published in English from January 2000 to May 2025 and studies involving human participants.

Search String

(Iatrogenic Injuries OR Inferior alveolar nerve OR "nerve injury" OR "nerve damage") AND (repair OR reconstruction OR neurolysis OR neurorrhaphy OR "nerve graft" OR "microsurgery") AND ("graft type" OR "donor site" OR "autograft" OR "nerve allograft" OR "vein graft" OR "sural nerve graft") AND ("postoperative complication" OR "neuropathic pain" OR "donor site morbidity" OR "functional sensory recovery" OR "FSR" OR "sensory improvement" OR "nerve regeneration").

Inclusion Criteria

Studies were included if they involved adults (≥18 years) with confirmed iatrogenic inferior alveolar nerve (IAN) injury directly attributed to dental or maxillofacial procedures such as third molar extraction, dental implant placement, orthognathic surgery, endodontic surgery, local anesthesia mishap, or iatrogenic mandibular fracture fixation. Only studies evaluating direct surgical nerve repair techniques explicitly performed in response to iatrogenic IAN injury were considered, including end-to-end microsurgical neurorrhaphy, autologous or processed nerve grafting, nerve conduit or tube use, tension-free nerve coaptation, and immediate or delayed surgical decompression intended as nerve repair. Studies comparing different surgical techniques showed that no repair, delayed repair, or pre- versus postoperative outcomes were eligible. Outcomes included functional sensory recovery, pain relief, sensory improvement, subjective sensory scores, light touch, trigeminal somatosensory evoked potentials, neurosensory function (Medical Research Council scale), visual analog scale, two-point discrimination, subjective sensation scores, patient satisfaction scores, edema, operative time, neural disturbances, implant stability (ISQ), marginal bone loss, Frankl's behavior score, implant survival, directional sense detection, touch thresholds, cold sensation response, and return of sensory function. Eligible study designs were randomized controlled trials, non-randomized controlled studies, and prospective or retrospective cohort studies ranging from January 2000 to May 2025. 

Exclusion Criteria

Studies were excluded if they involved non-iatrogenic nerve injuries (e.g., trauma, malignancy, radiation, congenital), did not identify the cause as iatrogenic, or had mixed etiologies without separate analysis of iatrogenic cases. Also excluded were studies focusing on adjunctive therapies without direct surgical repair, conservative or non-surgical management only, case reports, reviews, editorials, animal or cadaveric studies, and general nerve repair studies where iatrogenic IAN data could not be extracted.

Study Selection Process

The initial screening involved independent reviewers reading the articles' titles and abstracts. Then, the independent reviewers conducted a full-text review by comprehensively reading the articles. A consensus was reached regarding the reviewers' disagreement through discussion or the involvement of another reviewer. The review included only those studies available in full text that met the inclusion criteria.

Critical Appraisal Tool Used For Quality Assessment

The Joanna Briggs Institute (JBI) critical appraisal tools are used to assess the methodological quality and potential risk of bias in various research studies. Each tool contains a checklist of items typically ranging from eight to 13 questions, depending on the study design (e.g., randomized controlled trials and cohort studies). Each item is generally scored as "Yes" (1 point) or "No", "Unclear", or "Not applicable" (0 points). The JBI critical appraisal tool assesses study quality using a checklist scored as "Yes" (1) or "No/Unclear" (0). A score ≥70% indicates high quality, 50-69% moderate quality, and <50% low quality [17].

Data Extraction and Synthesis

A datasheet was created to collect details about the data we need to extract from the included studies to synthesize study findings. The current research encompasses basic information, such as author/year, study characteristics, causes of IAN injury, surgical techniques and outcomes, factors affecting outcomes, limitations, and challenges. After that, a thematic analysis using an inductive, data-driven approach was employed to analyze the data sheet. Then, an iterative approach was applied for a further in-depth study and convergence of the results [18]. Then, studies are analyzed critically to synthesize the evidence, ensuring the practice is evidence-based.

Ethical Consideration

**Figure 1:**
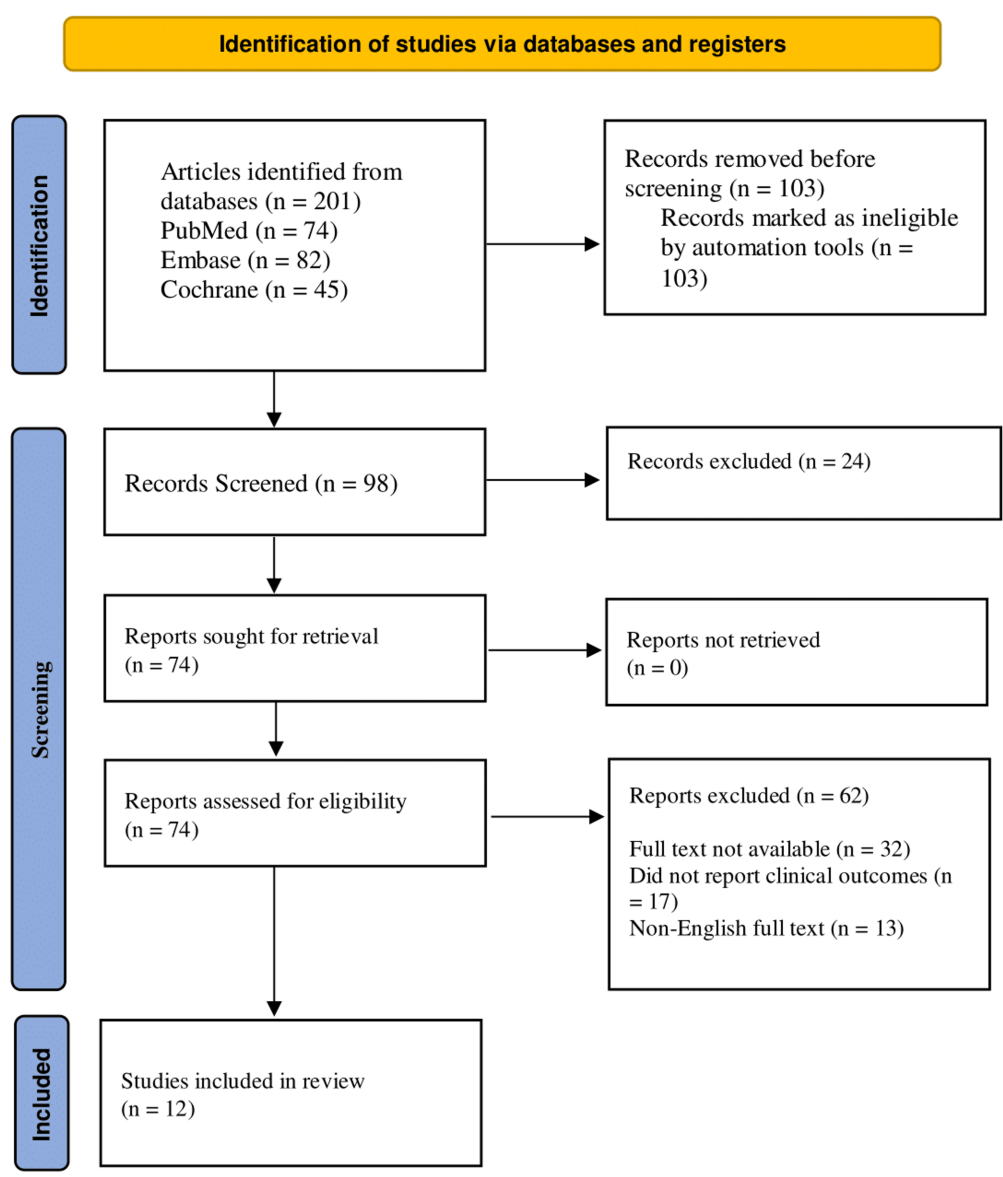
PRISMA flowchart PRISMA - Preferred Reporting Items for Systematic reviews and Meta-Analyses

The review adhered to the Helsinki Declaration to meet ethical standards throughout the study. There are no conflicts of interest among the reviewers. It was performed with specific keywords to reproduce it. The study will be published in a medical journal to disseminate findings publicly while ensuring confidentiality and anonymity. The study met the PRISMA guidelines (Figure 1).

Results

The PRISMA guidelines were followed to synthesize the evidence in this review systematically. The 201 articles were retrieved during the initial search using keywords, text words, and controlled vocabulary on PubMed, Embase, and the Cochrane Library databases. The 103 duplicate articles were removed. Then, 98 articles were selected for screening. Then, 24 irrelevant articles were removed based on title and abstract reading. Furthermore, 62 articles were removed based on non-retrieval of the studies, not reporting clinical outcomes, and non-English language articles. After that, only 12 articles were selected for quality assessment.

Critical Appraisal and Quality Assessment of Included Studies

The Joanna Briggs Institute (JBI) critical appraisal tools are used to assess the methodological quality and potential risk of bias in various research studies. Table 2 presents the JBI Critical Appraisal Checklist results for cohort studies, evaluating eleven key methodological criteria such as the similarity of groups, validity of exposure and outcome measurements, identification and management of confounders, follow-up completeness, and appropriateness of statistical analysis.

**Table 2:** JBI Critical Appraisal Checklist for cohort studies JBI - Joanna Briggs Institute

	Were the two groups similar and recruited from the same population?	Were the exposures measured similarly to assign people to both exposed and unexposed groups?	Was the exposure measured in a valid and reliable way?	Were confounding factors identified?	Were strategies to deal with confounding factors stated?	Were the groups/participants free of the outcome at the start of the study (or at the moment of exposure)?	Were the outcomes measured in a valid and reliable way?	Was the follow-up time reported and sufficient to be long enough for outcomes to occur?	Was follow-up complete, and if not, were the reasons for loss to follow-up described and explored?	Were strategies to address incomplete follow-up utilized?	Was appropriate statistical analysis used?	Total quality assessment score for each study	Quality Assessment
Bagheri et al. [19]	Yes	Yes	Yes	Yes	Yes	Yes	Yes	Yes	Yes	No	No	8/72.7%	High Quality
Biglioli et al. [20]	Yes	Yes	Yes	Yes	Yes	Yes	Yes	Yes	Yes	Yes	Yes	9/81.8%	High Quality
Byun et al. [21]	No	Yes	Yes	No	No	Yes	No	Yes	Yes	No	Yes	4/36.4%	Low Quality
Kang et al. [22]	Yes	No	Yes	Yes	Yes	Yes	No	Yes	Yes	No	Yes	6/54.5%	Medium Quality
Barbu et al. [23]	Yes	No	Yes	Yes	Yes	Yes	Yes	Yes	Yes	Yes	Yes	7/63.6%	Medium Quality
Robinson et al. [24]	Yes	No	Yes	Yes	Yes	Yes	No	No	Yes	Yes	Yes	6/54.5%	Medium Quality
Pogrel [25]	Yes	No	Yes	Yes	Yes	Yes	Yes	Yes	Yes	Yes	Yes	8/72.7%	High Quality
Pogrel and Maghen [26]	No	Yes	Yes	No	No	Yes	No	Yes	Yes	No	Yes	4/36.4%	Low Quality
Pitta et al. [27]	Yes	No	Yes	Yes	Yes	Yes	No	No	Yes	Yes	Yes	6/54.5%	Medium Quality

Based on the total number of "yes" responses, each study was scored and categorized by quality. Three studies by Bagheri et al., Biglioli et al., and Pogrel achieved high-quality ratings (scores ≥70%) [19,20,25], while two studies by Byun et al. and Pogrel and Maghen were rated low quality with scores below 50% [21,26]. The remaining four studies, including those by Kang et al., Barbu et al., Robinson et al., and Pitta et al., fell into the medium-quality category with scores ranging between 50% and 69% [22-24, 27].

Table 3 summarizes the JBI Critical Appraisal Checklist results for randomized controlled trials (RCTs), assessing 13 methodological criteria, including the use of proper randomization, allocation concealment, blinding, consistency of treatment delivery, follow-up completeness, outcome measurement reliability, and appropriateness of statistical analysis. Garoushi et al. demonstrated high methodological quality with a score of 76.9%, fulfilling most critical criteria [22]. Chehata et al. received a medium-quality rating of 61.5%, showing limitations, particularly in blinding and treatment consistency [28]. In contrast, Abdo et al. was rated low quality, scoring 46.2% because of shortcomings in blinding, follow-up analysis, and outcome measurement practices [22].

**Table 3:** JBI Critical Appraisal Checklists for randomized controlled trial

Checklist	Was true randomization used for the assignment of participants to treatment groups?	Was allocation to treatment groups concealed?	Were treatment groups similar at the baseline?	Were participants blind to treatment assignment?	Were those delivering treatment blind to treatment assignment?	Were outcomes assessors blind to treatment assignment?	Were treatment groups treated identically other than the intervention of interest?	Was follow up complete and if not, were differences between groups in terms of their follow-up adequately described and analyzed?	Were participants analyzed in the groups to which they were randomized?	Were outcomes measured in the same way for treatment groups?	Were outcomes measured in a reliable way?	Was appropriate statistical analysis used?	Was the trial design appropriate, and any deviations from the standard RCT design	Total quality assessment score for each study	Quality Assessment
Chehata et al. [28]	Yes	Yes	Yes	Yes	No	Yes	No	Yes	Yes	No	Yes	Yes	Yes	8/61.5%	Medium quality
Abdo et al. [29]	Yes	No	Yes	Yes	No	No	Yes	No	No	No	Yes	Yes	Yes	6/46.2 %	Low quality
Garoushi et al. [30]	Yes	Yes	Yes	Yes	Yes	Yes	Yes	Yes	Yes	Yes	Yes	Yes	Yes	10/76.9%	High quality

Characteristics and Findings of Included Studies ​​​​​​

Table 4 describes the characteristics and findings of studies included in the review. It encompasses author/year, objectives, research design/population characteristics, causes of IAN injury, surgical techniques, outcomes, factors affecting outcomes, limitations, and challenges.

**Table 4:** Characteristics and findings of studies included in the review IAN - inferior alveolar nerve; IANL - inferior alveolar nerve lateralization; IANB - inferior alveolar nerve block; SN - submental nerve; GAN - greater auricular nerve; VAS - visual analogue scale; PQ - pain questionnaire; LT - light touch; NST - neurosensory testing; TSEP - trigeminal somatosensory evoked potentials; MIR - mentalis intraoral reflex; PRF - platelet-rich fibrin; ICG - indocyanine green; ABBM - anorganic bovine bone mineral; ISQ - implant stability quotient; ILA - inferior labial artery; FSR - full sensory recovery; MRC - Medical Research Council; NSD - neurosensory disturbance

Author/Year	Study characteristics	Causes of IAN Injury	Surgical techniques	Outcome	Factors affecting outcomes	Limitations and challenges	
Bagheri et al. [19]	Cohort Study	Third molar removal (37.6%), mandibular SSRO (16.7%), dental implant placement (8.1%), endodontic treatment (7.5%)	1. External Decompression (n = 20): lateral unroofing of IAC	1. External Decompression: - 85% success (17/20) achieved FSR (S3-S4)	Positive Factors: - Early intervention (<12 months post-injury; OR 0.898/month, p<0.001) - Younger age (<51 years; OR 0.97/year, p=0.015)	Methodological: Retrospective design, Heterogeneous injury causes, No control group.	
	Population: 167 patients (126 female, 41 male), mean age: 38.7 years (range 15-75)						
			2. Internal Neurolysis (n = 60): epineurium opening, scar removal	2. Internal Neurolysis: 75% success (45/60) achieved FSR			
			3. Neuroma Excision (n = 17): removal of exophytic neuroma	3. Neuroma Excision: 70.6% success (12/17) achieved FSR	Negative factors: - Delay >12 months (threshold drop in success), Age >51 years		
			4. Neurorrhaphy (n = 18): Direct nerve suturing	4. Neurorrhaphy: 88.9% success (16/18) achieved FSR			
			5. Autogenous nerve graft (n = 71): Sural (SN) or great auricular (GAN) grafts	5. Autogenous nerve graft: 87.3% success (62/71) achieved FSR	Non-Significant Factors: - Etiology of injury (p=NS), Presence of pain (p=0.14) - Surgical technique (p=0.20)	Clinical: Donor site morbidity (temporary foot numbness with SN grafts), Technical difficulty of microsurgery, 18.3% of cases showed incomplete recovery.	
Biglioli et al. [20]	Cohort study	Endodontic treatment of mandibular molars or premolars	1. IAN Neurolysis (n = 2): Removal of endodontic material, Nerve decompression	Neurolysis: 50% (1/2) partial pain relief; both showed partial sensory improvement.	Positive Factors: Early intervention (<12 months), Sural nerve grafting, Complete removal of endodontic material	Methodological: Small sample size (n=7), No control group, Subjective pain assessment.	
	Population: 7 female patients (mean age 46, range 35-68)		2. Sural nerve graft (n = 4): - Resection of the damaged segment - 2-5 cm graft from sural nerve - Microsurgical repair	Sural nerve graft: 100% (4/4) pain relief, Best sensory recovery (though incomplete), Donor site morbidity (temporary foot numbness)			
					Negative Factors: Delayed treatment (>12 months), Vein grafting, and severe initial nerve damage.	Clinical: Donor site morbidity, Incomplete sensory recovery in all cases. Technical difficulty of microsurgery.	
Byun et al. [21]	Cohort Study	Endodontic overfilling of calcium hydroxide paste during root canal treatment of mandibular molars/premolars,	Foreign body removal	Overall, 7/9 patients achieved FSR	Positive: Early intervention, localized nerve damage.	Delayed referrals - Difficulty in removing widely spread material.	
	Population: 9 patients (7 female, 2 male; mean age 36.3 years)		Microsurgical decompression (external/internal)	Early repair (<60 days): 3/5 achieved FSR (mean time: 198 days).	Negative: Widespread nerve injury (poor outcomes with SSRO), delayed referral.	Legal implications affecting subjective reporting.	
			Excision of neuroma + nerve repair	Late repair (>60 days): 4/4 achieved FSR (mean time: 241 days).			
			Excision + interposition sural nerve graft				
				Significant improvements in neurosensory tests (p-values: 0.01–0.048).			
			Surgical approaches: Decortication, lateral window, and sagittal split osteotomy (SSRO).				
				VAS: 4 patients reported significant improvement, 3 mild, and 2 no improvement.			
Kang et al. [22]	Cohort study	Dental implant placement (93.8%)	Nerve Sliding Technique (NST): Intentional transection of the incisive branch. Posterior relocation of the distal IAN stump. Direct epineural anastomosis with 10-0 nylon. Allogenic bone grafting for bony defects d Suction drain placement.	62.5% achieved FSR (S3+ or higher on the MRC scale). Median time to FSR: 84.5 days. Early repair (≤2 months): 85.7% FSR vs. 44.4% in late repair (>2 months; p=0.068). No FSR after 1 year post-op	Positive: Younger age (p=0.041), absence of dysesthesia (p=0.019).	Technical: Limited to gaps ≤15 mm; precise mobilization of nerve stumps required.	
	16 patients (mean age: 56.1 ± 10.1 years; 75% female)				Neutral: Gender, neuroma formation.	Clinical: Dysesthesia reduced FSR likelihood.	
					Negative: Dysesthesia, delayed repair (>2 months).	Limitations: Small sample size; no comparison to autografts/conduits.	
Barbu et al. [23]	Cohort study	Dental implant placement	Modified IAN repositioning: Lingual shift of muco-periosteal incision. Smaller osteotomy window (5–6 mm). Ultrasonic bone surgery (Piezosurgery). Simultaneous dental implant placement (32 implants).	Implant Survival: 100% (no loss during follow-up).	Positive: Ultrasonic bone surgery reduced thermal damage.	Anatomical: Mental foramen located on residual crest, risking nerve injury.	
	7 patients (11 procedures; mean age 43.29 years)			Neurosensory outcomes: Transient neural disturbances in all patients (resolved within 2 months). No permanent neurosensory deficits.	Negative: Extreme bone resorption and minimal keratinized tissue required modified techniques.	Surgical: Precise osteotomy needed due to minimal bone height.	
				Follow-up: Mean 35.71 months (7–120 months).		Postoperative: Managing transient neurosensory disturbances.	
Robinson et al. [24]	Cohort study	Third molar extractions (18 patients), dental implant placement (2 patients), and cyst removal and mandibular fracture management	Inferior alveolar nerve decompression and neurolysis: Intraoral approach with buccal plate removal, Microscopic neurolysis (epineurium incisions), Limited neurorrhaphy (8/0 sutures in 6 patients), and Implant removal if causative (1 patient).	Neurosensory Outcomes: Pain Reduction: 64% → 36% (p=0.048), Tingling (VAS): 27% reduction (p=0.009)	Positive Factors: Radiographic evidence of canal disruption - Severe symptoms.	Methodological: Heterogeneous injury timing (2-96 months), No control group.	
	Population: 25 patients (21 female, 4 male) Mean Age: 45 years (range 22-62)			Subjective Sensation: 35% → 56% (p=0.01), Light Touch/Pinprick: Significant improvement (p=0.003 / p=0.015), two-point discrimination: Lip: 11.2→8.4mm (p=0.006); Chin: 13.3→11mm (p=0.03)	Negative Factors: Late intervention (>12 months post-injury), Female gender (84% of cohort).	Clinical: 16% developed new hypersensitivity, Variable individual outcomes, Incomplete recovery (all patients had residual numbness).	
				Patient Satisfaction: Median subjective value: 7/10 (range 0-10)	Non-Significant Factors: Age (p>0.05), Etiology of injury (p>0.05).		
Pogrel [25]	Retrospective study	Third molar extractions, dental implant placement	Decompression (5 patients), Direct anastomosis (26 patients)	Good improvement: 10 patients, Some improvement: 18 patients, No improvement: 22 patients, Worse: 1 patient (No statistical significance due to small sample size).	Early repair (<10 weeks): Better outcomes (6 good, 2 some improvement).	Subjective vs. objective outcome mismatch. Small sample size limits statistical power.	
	880 patients referred for inferior alveolar/lingual nerve injuries over 5 years		Nerve gap reconstruction: Vein graft (16), Gore-Tex tube (2), Autogenous nerve graft (2)		Nerve type: Inferior alveolar nerve repairs slightly better than lingual nerve (bony canal guidance).	Late repairs complicated by neuroma formation/distal stump degeneration. Insurance/patient delays in surgery timing.	
					Graft type: Direct anastomosis > vein graft > Gore-Tex tube.		
Pogrel and Maghen [26]	Cohort Study	Third molar surgery (14 cases), sagittal split osteotomy (1 case), and endodontic treatment (1 case)	IAN: Facial vein grafts (extraoral, microscope-assisted). Technique: Vein dilated, slid over nerve ends, secured with 8-0 nylon sutures.	IAN: ≤5 mm gaps: 2/3 cases good return, 1 partial: >5 mm gaps: 1/3 good return, 2 partial.	Gap length: Critical for success (≤5 mm optimal).	Valve interference: Saphenous vein valves hindered regeneration.	
					Nerve type: IAN had better outcomes due to canal stability.		
	(n=15 patients, 16 grafts)				Vein characteristics: Valve presence (saphenous) and mobility (lingual) impacted results.	Limited graft durability: Unsuitable for gaps >5 mm in lingual nerves.	
						Dysesthesia risk: 1 IAN case required neurectomy due to recurrent dysesthesia.	
Pitta et al. [27]	Cohort Study	Endodontic treatment (33%) and third molar removal (50%)	Gore-Tex (GT) tubing conduit: 3 mm diameter, 1–5 cm length. Injected with the patient’s serum. Sutured to epineurium with 9-0 nylon. Used for defects >1 cm.	Pain relief: 4/6 patients reported no change; 2/6 had minimal reduction (not clinically significant).	Negative: Long delay to repair (>3 months), significant defects (>1 cm), traumatic neuroma presence.	Technical: GT tubing collapse/fibrous ingrowth impeded regeneration.	
	6 patients (5 female, 1 male)			Sensory recovery: Touch: 3/6 responded (abnormal thresholds). Cold: 2/6 delayed response. Directional sense: 1/6 detected. 2-point discrimination: 3/6 responded (>20 mm vs. 6 mm normal).	Neutral: Age, gender.	Clinical: Poor access to trigeminal nerve branches; erratic outcomes.	
				No patient achieved functional sensory recovery (FSR).	Positive: None identified.	Limitations: Small sample size, no control group, heterogeneous injury timing/etiology.	
Chehata and Abdelmonim [28]	Randomized controlled trial	Dental implant placement	Group A (Piezosurgery): IAN lateralization using a piezoelectric device with PRF-coated implants.	Neurosensory recovery: Group A showed significantly better results at 2 weeks (p < 0.0001) and 8 weeks (p < 0.0001) via subjective tests (e.g., Two-Point Discrimination, Light Touch).	Technique precision: Piezo surgery minimized thermal/mechanical trauma to IAN.	Surgical complexity: IAN exposure risks (stretching/vascular damage).	
						Cost/equipment: Piezosurgery requires specialized devices.	
			Group B (Conventional): IAN lateralization using rotary surgical burs with copious irrigation. Both groups followed standardized osteotomy protocols.	Pain/edema: Lower VAS scores (p≤0.05) and edema (p≤0.05) in Group A at all intervals (2, 5, 7 days).	Bone quality: Atrophic mandibles required careful osteotomy planning.	Operative time: Piezosurgery prolonged procedure duration.	
					Patient age: Mean age ~55 years (potential slower nerve recovery).	Subjective bias: Patient-reported outcomes (e.g., VAS) may vary.	
				Operative time: Longer in Group A (p≤0.05). TSEP (Objective): Group A had shorter N-peak latency (p=0.0044 at 2 weeks) and higher N-P amplitude (p=0.0032), indicating less nerve damage.	Negative: Longer duration (>6 months) reduced subjective improvement (VAS).	Limitations: No control group; heterogeneous etiologies.	
Abdo et al. [29]	Randomized controlled trial	Implant placement	1. Inferior alveolar nerve lateralization (IANL) (Group A): Piezo surgery osteotomy nerve retraction + implant placement medial to IAN, PRF used.	IANL (Group A): Subjective Tests (PQ): Significant dysfunction at 2W (1.54/5) and 8W (2.46/5); recovery by 24W (4.54/5, p=0.09 vs. IANB), Light Touch (LT): 100% dysfunction at 2W, 38.5% at 8W, 23% at 24W - TSEP (N-peak latency): Prolonged latency at 2W (+2.71 ms, p=0.049)	Positive Factors for IANB: Computer-guided precision (2 mm from IAC), Minimal nerve manipulation	Methodological: Small sample size (n = 26), Single-center study	
	Population: 26 patients (13 per group), mean age: Group A (IANL): 53.46 ± 4.94; Group B (IANB): 56.66 ± 3.45		2. Inferior alveolar nerve bypass (IANB) (Group B): Computer-guided stent (On Demand 3D software), Implant placed 2 mm from IAC with 1 mm bucco-lingual bone.	IANB (Group B): Subjective Tests (PQ): Minimal dysfunction (3.62/5 at 2W, 4.38/5 at 8W, 4.85/5 at 24W) Light Touch (LT): 38.5% dysfunction at 2W, 8% at 8W, 0% at 24W, TSEP (N-peak latency): No significant latency changes (p>0.05)	Negative factors for IANL: Direct nerve retraction, Initial latency prolongation (TSEP).	Clinical: Wound dehiscence in 7.69% of IANL cases, Subjective test variability, Technical complexity of computer-guided planning	
					Non-significant factors: Age (p=NS), Bone width (5-6 mm required for IANB).		
Garoushi et al. [30]	Randomized clinical trial	Dental implant placement	1. Control group (n=15): Conventional IANL (Piezo surgery osteotomy)—IAN repositioned directly over implants	Frankl’s behavior score: No significant difference between groups (p=0.16).	Behavior: Negative behavior correlated with longer procedure time (p=0.053).	Methodological: Small sample size (n=18 patients), Short follow-up (6 months).	
				Neurosensory function (MRC Scale): Control: 66.7% S2 at 2W; 100% S4 at 6M, Test: 66.7% S2 at 2W; 100% S4 at 6M. No significant difference (p>0.05)	Positive factors: Bicortical implant engagement (primary stability), Piezo surgery reduced thermal trauma.		
	Population: 18 patients (12 males, 6 females), mean age: Control: 39.3 ± 7.9 yrs; Test: 40.7 ± 7.2 yrs 30 IANL procedures			Implant stability (ISQ): Control: 66.40→74.73 (p<0.001), Test: 65.60→74.73 (p<0.001), No intergroup difference (p=1.000).	Negative factors: Age (>40 yrs associated with greater bone loss, p=0.009), No benefit from collagen membrane/bone graft.	Clinical: 1 implant perforation (test group), Transient NSD in all patients, Technical complexity of graft/membrane placement.	
			2. Test group (n=15): IANL + collagen membrane isolation, Interposed bone graft (autogenous + ABBM) over implants.	Marginal bone loss: control: 0.38 ± 0.14mm, test: 0.42 ± 0.09mm. No difference (p=0.401)	Non-significant factors: gender (p=1.000), Surgical side (p=0.256).		

Study Characteristics

Summary of study designs: The systematic review included 12 studies, such as nine cohort studies and three randomized controlled trials (RCTs). Most of the studies were cohort studies that looked at the results after nerve decompression, neurorrhaphy, and nerve grafting surgeries. In some RCTs, inferior alveolar nerve lateralization (IANL) was set against nerve bypass (IANB), whereas earlier studies measured long-term results after various nerve repair techniques. Since the studies used a range of designs, it was possible to review nerve repair methods extensively, even with different methodological qualities among studies.

Population demographics: The studies used populations with various characteristics, and sample groups contained 6 to 167 patients. The participants' average age was generally somewhere between 36 and 56 years, and the majority were female in a number of studies. Bagheri et al. 2012 had 167 patients with a mean age of 38.7 years, whereas Kang et al. observed 16 subjects with a mean age of 56.1 years [19, 22]. Because populations differ in their health profiles, the studies addressed many types of medical issues.

Methodological rigor: The study quality was measured using the critical appraisal tools from the Joanna Briggs Institute (JBI). Research by Bagheri et al., Biglioli et al., Robinson et al., and Pogrel, scoring at least 70%, was considered highly reliable because of its strong design, proper handling of confounders, and trustworthy outcome counts [19,20,24,25]. Medium-quality studies (including those of Kang et al. and Barbu et al.) scored between 50% and 69%, as they had issues like a small number of participants or incomplete follow-ups [22, 23]. Low marks for some studies, such as Byun et al. and Pogrel Maghenn, often resulted from participants being similar between groups and the inability to blind the people involved adequately [21, 26]. It also pointed out that more robust RCTs are required to support evidence-based suggestions.

Cause of Inferior Alveolar Nerve Injury

Common surgical causes: The studies found that injury to the inferior alveolar nerve (IAN) most often happens as a result of the following surgical procedures: Many studies point to third molar extractions as the significant cause of IAN injuries. For example, Bagheri et al. reported that 37.6% of cases of IAN injuries resulted from third molar surgery [19]. Pogrel and Pogrel & Maghen point out that removing wisdom teeth is a major cause of nerve damage [25,26]. Several studies, for example, Kang et al. and Barbu et al., have documented cases where dental implants damaged the IAN, particularly in patients with low mandible bone or erroneous implant placements [22, 23]. Byun et al. found that overfilling calcium hydroxide during root canals created a problem, while Biglioli et al. identified injuries in mandibular molars after endodontic procedures [20,21]. Bagheri et al. mentioned that sagittal split ramus osteotomy (SSRO) is a condition that can lead to damage to the IAN [19].

Non-surgical causes and risk factors: IAN injury caused by things other than surgery was less commonly discussed, and Robinson et al. mentioned mandibular fracture as a reason for IAN injury [24]. According to several studies, including those by Bagheri et al. and Byun et al., getting treatment late (over 12 months after the injury) reduces recovery success [19, 21]. Barbu et al. pointed out that having the mental foramen close to the residual crest and severe jaw shrinkage increases the risk [23]. Bagheri et al. and Biglioli et al. reported that older patients (above 51 years) and cases of severe nerve damage are often linked to a poor outcome [19, 20].

Surgical Techniques For Nerve Repair 

Decompression, neurolysis, grafting, and neurorrhaphy are surgical options for iatrogenic inferior alveolar nerve (IAN) injuries, according to the comprehensive review. Decompression often relieves nerve pressure by removing adjacent bone or scar tissue, as shown in investigations by Bagheri et al. and Robinson et al. [19,24]. Biglioli et al. [20] acknowledge that neurolysis, whether inside or outside, can provide mixed consequences in nerve release. Nerve grafts from patients' own nerves, including sural or greater auricular, have good success rates for wider gaps, as confirmed by Bagheri et al. and Kang et al. [19,22]. Pogrel [25] reported that neurorrhaphy, which involves direct nerve mending, worked for clean nerve transections when no tension was left.

Comparing techniques: Nerve injury kind and severity help to determine the surgical technique. Decompression and neurolysis are less invasive and more effective in early-stage injuries with modest nerve injury, as Robinson et al. showed that 85% of patients recovered fully sensorially [24]. Patients with substantial injuries or nerve gaps do not benefit from these treatments. Sural nerve grafts had a high success rate, 87.3% FSR in Bagheri et al., but could cause temporary numbness [19]. According to Pogrel [25], neurorrhaphy was successful for direct repairs but required perfect microsurgery and tension-free nerve alignment. The effectiveness of advanced tools like those indicated by Kang et al. and Abdo et al. was limited by their complexity and limited patient evaluation [22, 29]. According to Chehata et al., piezoelectric surgery caused less heat damage than usual procedures [28]. The vascular grafts provided by Pogrel & Maghen could not cover gaps above 5 mm due to their design [26]. According to Byun et al. in 2015 [21], success depended on responding promptly, contacting nerves minimally, and choosing the best grafts. Delays or substantial nerve injury frequently resulted in poor outcomes.

Outcomes of Nerve Repair Procedures

Functional sensory recovery (FSR): Recovery of sensation through surgeries differed according to the technique applied. The success rate for external decompression was higher at 85% (17 out of 20 patients reached FSR), compared to internal neurolysis at 75% (45 out of 60 patients). Among patients with neurorrhaphy, 88.9% (16/18) had positive outcomes, and patients who received autogenous nerve grafts (e.g., sural or great auricular) reached an 87.3% positive outcome (62/71), as indicated by Bagheri et al. [19]. The authors found that the nerve sliding technique resulted in a success rate of 62.5%, and if surgery was done early (within two months), the success rate was 85.7%, while late repairs (after two months) had an FSR of just 44.4%, according to Kang et al. [22]. 100% of patients in the Biglioli et al. study had their pain and sensory function restored after receiving a sural nerve graft [20]. Even so, Pitta et al. noted that Gore-Tex tubing failed to produce encouraging FSR outcomes [27].

Pain and sensory relief: Pain reduction and sensory improvement were notable outcomes across studies. External decompression and neurolysis significantly reduced pain and tingling, with Robinson et al. reporting a 64% reduction in pain and a 27% improvement in tingling sensations. Sural nerve grafts provided pain relief in all cases [20]. Piezosurgery for nerve lateralization resulted in lower pain scores (VAS) and edema compared to conventional methods, with subjective tests showing significant improvements in sensory function [28]. However, delayed interventions (>12 months post-injury) were less effective, with Pogrel noting that only 10 out of 50 patients reported good improvement in sensory function [25].

Recovery times: Recovery times varied by technique and timing of intervention. Early repair (<12 months post-injury) was associated with faster and more complete recovery, with Bagheri et al. noting higher FSR rates for early interventions. Kang et al. reported a median recovery time of 84.5 days for the nerve sliding technique, with early repairs achieving FSR in 85.7% of cases [22]. Byun et al. found that early microsurgical decompression (<60 days post-injury) led to FSR in 3/5 patients (mean time: 198 days), while late repairs (>60 days) took longer (mean time: 241 days) [21]. Computer-guided nerve bypass (IANB) showed faster subjective recovery at 24 weeks than nerve lateralization (IANL). However, both methods required prolonged follow-up for complete assessment, as indicated by Abdo et al. [29].

Factors Influencing Outcomes

A patient's age and intervention time are important influences on the results. Studies by Bagheri et al. and Kang et al. show that the younger a patient and the earlier the intervention takes place, the more likely they are to be successful [19, 22]. Treatment delayed for over one year is usually more likely to result in less sensory improvement. Gender and the extent of the initial injury are essential, as having severe nerve damage or injury throughout the body makes for less favorable outcomes, as indicated by Byun et al. [21]. In Bagheri et al., external decompression and direct neurorrhaphy had success rates of 85% and 88.9%, respectively [19]. Sural nerve grafts gave better results (87.3% successful) than vein grafts or Gore-Tex conduits, as indicated by Pogrel & Maghen [26]. Piezosurgery was shown in the study by Chehata et al. to reduce thermal damage and better recovery of sensation when compared to conventional approaches [28]. If surgery uses precise computer-guided methods followed by proper care, results are more positive, according to Abdo et al. [29]. Detecting problems early and aiming for minimal touch on nerves during surgery is essential. Furthermore, discomfort or nerve problems at the donor site (e.g., numbness following a sural nerve graft) can change patient satisfaction and recovery, as described by Biglioli et al. [20].

Discussion

The systematic review includes a total of 12 studies (nine cohorts and 30 RCTs), including sample sizes of 6-16 patients, with the majority of patients being adult females. The most common iatrogenic IAN causes are third molar extractions and dental placements, accounting for around more than 70% of the included studies' cases. The review identified neurorrhaphy, sural nerve grafting, external decompression, and internal neurolysis, with their associated functional sensory recovery (FSR) rates ranging from 88.9% to 60%. Neurorrhaphy (direct nerve repair) showed the highest success rate with a mean FSR of 88.9%; Sural nerve grafting demonstrated an FSR of 87.3%; the external decompression technique had an FSR between 70% and 82%; and internal neurolysis utilized for complex cases showed the lowest mean FSR of around 60% with longer recovery periods. Moreover, it is determined that the timing of intervention (early intervention) was a key determinant of the success rate of intervention. Surgical repair within two months was related to a significantly higher FSR (mean 84.2%) than surgical repair after six months, which had a mean FSR reduced to 57.1%. Hoshino et al. also examined that early intervention also decreased the risk of permanent paresthesia by one-third (30%). Therefore, early and targeted intervention showed the most favorable outcomes. The review also highlighted that the use of piezo surgery minimized thermal damage and reduced nerve injury risk, reporting only a 3.3% incidence of transient paresthesia compared to 13.5% during conventional rotary treatments. However, variability in outcomes such as age, nerve injury severity, surgical expertise, and the presence of high heterogeneity in study design and outcome assessment limited the direct comparison of nerve repair strategies to determine the true superiority of the intervention to manage inferior alveolar nerve injury (IAN).

The review identified that early surgical intervention is crucial because alveolar nerve surgical repair within two months was related to a significantly higher FSR (mean 84.2%) than surgical repair after six months, with a mean FSR reduced to 57.1% after six months. These findings also support the principle that delayed nerve repair may lead to Wallerian degeneration and poorer outcomes due to fibrosis and irreversible axonal loss [31]. The findings are consistent with the study conducted by Miloro et al., who reported that alveolar nerve repair with allograft showed FSR rates of above 80%, but delayed repairs after six months yielded an FSR of 57% [32]. Neurorrhaphy (direct nerve repair) showed the highest success rate with a mean FSR of 88.9%, and sural nerve grafting demonstrated an FSR of 87.3%. These findings are consistent with the study conducted by Zuniga, who performed direct neurorrhaphy to repair the transected alveolar nerve with an allograft and examined functional sensory recovery of 87% to 100% [33].

Moreover, nerve grafting is recommended when there is a gap between the injured nerve ends. Yépez et al. also reported that allograft and autogenous grafts showed similar FSR rates ranging between 93.75% (less than 1 cm gap) and 78.9% (>1 cm gap) [34]. Decompression and neurolysis have shown moderate effectiveness, ranging from 70% to 82%. Decompression is less effective in longstanding and severe injuries, but neurolysis is utilized for complex cases due to the risk of trauma. Moreover, the literature also demonstrates that the selection of the intervention for inferior alveolar nerve repair depends upon the patients' related factors, type of injury, and severity [19]. Therefore, the timing and selection of surgical repair techniques should be personalized. Innovative and minimally invasive techniques such as Piezo surgery and computer-assisted surgical planning showed promising results. Piezo surgery significantly reduced iatrogenic nerve damage, lowering the incidence of transient paresthesia to just 3.3% of cases. Moreover, computer-assisted surgical planning, such as IAN bypass, enhances surgical precision and safety. However, the data reported are limited and require long-term positive outcomes validation with longitudinal study designs. Therefore, future researchers should consider these techniques and compare them for the treatment of iatrogenic IAN injuries.

Some of the reported challenges in nerve repair strategies include autogenous nerve grafts, like the sural nerve, often causing donor site morbidity (5-12%), including temporary foot numbness, though symptoms typically resolve over time. Surgical challenges include precise nerve mobilization, microsurgical anastomosis, and osteotomy accuracy, highlighting technical difficulties that emphasize the need for specialized skills. Incomplete recovery remains an issue, with residual numbness or dysesthesia reported, particularly in delayed or severe cases, underscoring the importance of early intervention for better outcomes. These challenges need solutions in future research to address them comprehensively for the benefit of patients in clinical settings. The review has strengths, such as including randomized controlled trials and cohort studies to synthesize evidence, which increased the evidence quality. The JBI critical appraisal tool was used for methodological quality assessment, which reported moderate- to high-quality evidence. The review identified a variety of surgical repair strategies that allow a multifaceted understanding of surgical repair techniques, considering personalized factors. Moreover, the sample size is adequate and was obtained from primary study designs whose findings are clinically applicable with some caution because of the varying moderate to high evidence quality, requiring further validation of findings.

However, there are some limitations of this systematic review, such as heterogeneity in study designs, surgical interventions, and outcome measures, and factors responsible for disease and modifying factors were not adequately addressed in the included studies, which restricts the direct comparison of surgical interventions and meta-analysis. The adequate sample size also restricted the findings' generalizability. The variable follow-up duration in each study also underestimates long-term complications and late recoveries. There is also a lack of standardization in reporting outcomes, particularly for subjective versus objective sensory tests.

The implication of this systematic review is that early intervention, particularly within two months, may improve the patients' clinical outcomes. The selection of intervention and timing is crucial. Moreover, the selection of nerve repair strategies such as neurorrhaphy, grafting, decompression, and internal neurolysis also depends upon patient-related factors, injury severity, and the timing of repairing the injury. The review also highlights the need for patient-specific surgical planning, particularly among older adults and complex cases. The review also supports the development and adoption of less invasive, thermally safe tools such as piezo surgery. The systematic review recommends that future researchers conduct large, multicenter RCTs, comparing nerve-repairing techniques with standardized guidelines. Future researchers must focus on devising standardized outcome measure tools that help to compare and draw a consensus on the superior role of each intervention. 

## Conclusions

The review highlighted that early surgical intervention is a key determinant in achieving optimal results for IAN injury treatment. The review observed findings when surgical strategies are performed within two months of the incident because this greatly improves the chances of regaining functional sensory recovery in the affected area. The highest success rate was found with direct neurorrhaphy and sural nerve grafting among a variety of surgical techniques, most noticeable in clean injuries. The decompression and neurolysis techniques showed modest effectiveness, although they worked better in a few chronic compression cases. New approaches like using piezosurgery and computers to help with surgical navigation improve safety and lessen the injury caused by surgery. Nevertheless, nerve grafting usually leads to some short-term and easy-to-handle donor site morbidity. The lack of standardized outcome reporting and direct comparative randomized controlled trials with homogenous assessment tools limits the generalizability of the findings and recommends further validation. Finally, individualized treatment planning based on the timing of intervention, injury characteristics, and patient-centered factors is vital for optimizing IAN recovery.
